# Intraoperative Monitoring Cerebral Blood Flow During the Treatment of Brain Arteriovenous Malformations in Hybrid Operating Room by Laser Speckle Contrast Imaging

**DOI:** 10.3389/fsurg.2022.855397

**Published:** 2022-05-06

**Authors:** Sicai Tao, Tingbao Zhang, Keyao Zhou, Xiaohu Liu, Yu Feng, Wenyuan Zhao, Jincao Chen

**Affiliations:** ^1^Department of Neurosurgery, Zhongnan Hospital, Wuhan University, Wuhan, China; ^2^Britton Chance Center for Biomedical Photonics, Wuhan National Laboratory for Optoelectronics, Huazhong University of Science and Technology, Wuhan, China

**Keywords:** cerebral blood flow, Intraoperative cortical perfusion imaging, laser speckle contrast imaging, brain arteriovenous malformations, hemodynamics, hybrid surgery, preoperative embolization

## Abstract

**Background:**

Hemodynamic changes caused by hybrid surgery for brain arteriovenous malformations (bAVM) are usually related to long-term lesions from “blood stealing”. There are currently no viable low-cost, noninvasive procedures for assessing cerebral perfusion in the operating room. This study aims to investigate the use of intraoperative laser speckle contrast image (LSCI) software in AVM surgery.

**Methods:**

In Zhongnan Hospital of Wuhan University, 14 patients who underwent surgery with LSCI were collected. To analyze the hemodynamic features of AVM and the influence on the peripheral cortex of AVM embolization and resection, we assessed the transit time between feeding arteries and drainage veins by intraoperative digital subtraction angiography (DSA). Meanwhile, LSCI was performed at pre-embolization, post-embolization, and after complete resection of bAVM.

**Results:**

In this study, the transit time of bAVM before and after embolization was compared, the transit time before embolization was significantly shorter than that after embolization (*p* < 0.05). We also got good visualization of relative CBF, in addition, to flow imaging in the cortical vasculature round bAVM with LSCI. The flux of post-surgery was significantly higher than pre-embolization (*p* < 0.01).

**Conclusion:**

Hemodynamic variable assessment plays an important role in the resection of AVM in the hybrid operative room and LSCI can be used to visualize and evaluate cortical cerebral blood flow to detect pathological hyperperfusion in real-time with a good spatial-temporal resolution in a sensitive and continuous, non-invasive mode.

## Introduction

The treatment of complex brain arteriovenous malformations (bAVM) remains a challenge for neurosurgeons (1, 2). In the past decades, we have learned more about the anatomy of bAVM and made a great advance in therapy (2–5). Complete nidus obliteration is the only therapeutic treatment, while partial resection is neither efficacious nor prevents recurring bleeding (5–10). In recent years, the combination of intravascular therapy and microsurgery in the hybrid operating room (hybrid-OR) seems to benefit the majority of patients by increasing the occlusion rate (1, 3, 6, 10–13).

Approximately 3–12.5% of patients with bAVM undergoing microsurgery have intracerebral hemorrhage (ICH) or severe edema, which may be caused by residual lesions or hemodynamic abnormalities after bAVM resection (14). Cerebral hyperperfusion plays an important role in the postoperative complications of bAVM. Cerebral hyperperfusion, which is characterized by a rapid increase in regional cerebral blood flow (CBF), results in the interruption of the automatic regulation function of the cerebrovascular system (15). Therefore, real-time vascular perfusion imaging and hemodynamic monitoring have become critical components of the excision of bAVM (5, 16).

Although indocyanine green videoangiography (ICG) (5, 17, 18) and intraoperative digital subtraction angiography (DSA) (13, 18) are able to assess arterial blood flow during neurosurgery, microcirculation, or cerebral perfusion cannot be showed. Laser speckle contrast imaging (LSCI) allows semiquantitative imaging of tissue perfusion (19–22). The lens radiates the laser to illuminate the investigated area, and the charge coupled device (CCD) camera captures the illuminated area in real time to create a two-dimensional color-coded blood flow map (23). Hecht et al. (20) demonstrated that intraoperative LSCI is feasible, safe, and effective in the process of neurosurgery.

This study was aimed to demonstrate hemodynamic changes after complete resection of bAVM and highlight the effectiveness and availability of intraoperative LSCI, which could continuously monitor normal cortical CBF during hybrid surgery in real-time.

## Methods

### Patients

The study was approved by the local ethics committee of the (Zhongnan Hospital, Wuhan University) and a total of 14 patients with bAVM were admitted from May 2020 to October 2021. The data of the patients are shown in Table 1. All patients with hybrid surgery underwent bAVM embolization and microsurgical resection under general anesthesia according to the fixation scheme.

**Table 1:** Summary of patients with AVM.

**Patient**	**Age**	**Sex**	**Presentation**	**S-M grade**	**Location**	**Size/cm**	**No of LSCI**	**Transit time/ms**		**Operative time/h**	**Blood loss/ml**	**GCS of 7 days after operative**	**mRS**
								**Before**	**After**				
1	44	M	Epilepsy	4	L, T	6.0*4.5*3.0	3	1,333	667	8	1,000	14	0
2	49	F	Neurological deficits	3	L, T	5.4*3.1*3.5	3	1,167	1,667	12	1,000	14	0
3	45	M	Epilepsy	3	R, F	5.0*3.0*3.5	3	1,333	2,167	7	100	13	0
4	38	M	Headache	5	L, P	6.5*7.0*4.5	3	1,000	1,333	12	1,500	15	0
5	16	F	Headache	3	R, F	5.0*3.5*3.0	3	1,667	2,333	6	300	15	0
6	25	F	Epilepsy	4	R, F	3.5*6.0*4.0	3	667	1,667	7	200	15	0
7	30	M	Headache	4	R, O	6.0*4.0*3.0	3	1,333	1,333	8	400	13	1
8	31	M	Headache	4	L, F	5.5*4.0*3.5	3	1,000	1,333	6	400	13	0
9	28	M	Headache	3	R, TO	4.0*3.5*3.0	3	1,000	1,667	9	1,500	11	2
10	11	F	Epilepsy	4	R, TO	5.0*4.0*4.0	3	1,667	1,500	11	800	15	0
11	10	M	Hemorrhage	4	L, Cerebellar	4.5*4.0*3.5	3	1,333	1,833	11	5,000	15	0
12	59	M	Hemorrhage	4	L, PO	6.0*5.0*3.5	3	667	1,667	10	7,000	15	0
13	53	M	Epilepsy	4	R, T	3.0*4.0*4.5	3	1,167	1,000	7	500	14	0
14	49	M	Epilepsy	3	R, T	3.5*4.0*3.0	3	1,000	667	6	600	15	0

*P, parietal lobe; F, frontal lobe; O, occipital lobe; T, temporal lobe*.

### Laser Speckle Contrast Imaging

After imaging is illuminated by laser light, a random interference phenomenon called laser speckle occurs. The random speckle pattern, which is exploited by laser speckle contrast analysis, can be generated by irradiating the tissue with laser light and varies as blood cells move in the selected area (23, 24). When there is a high level of movement (fast flow), the dynamic pattern becomes more and more blurry and the contrast in the area decreases. As a result, low contrast is associated with high flow, while high contrast is related to low flow. The contrast image is processed to provide a color coded real-time image (flux image: red = high flow, blue = low flow), which is consistent with the blood flow velocity in the tissue, commonly referred to as “flux” (20, 25).

**Figure 1:**
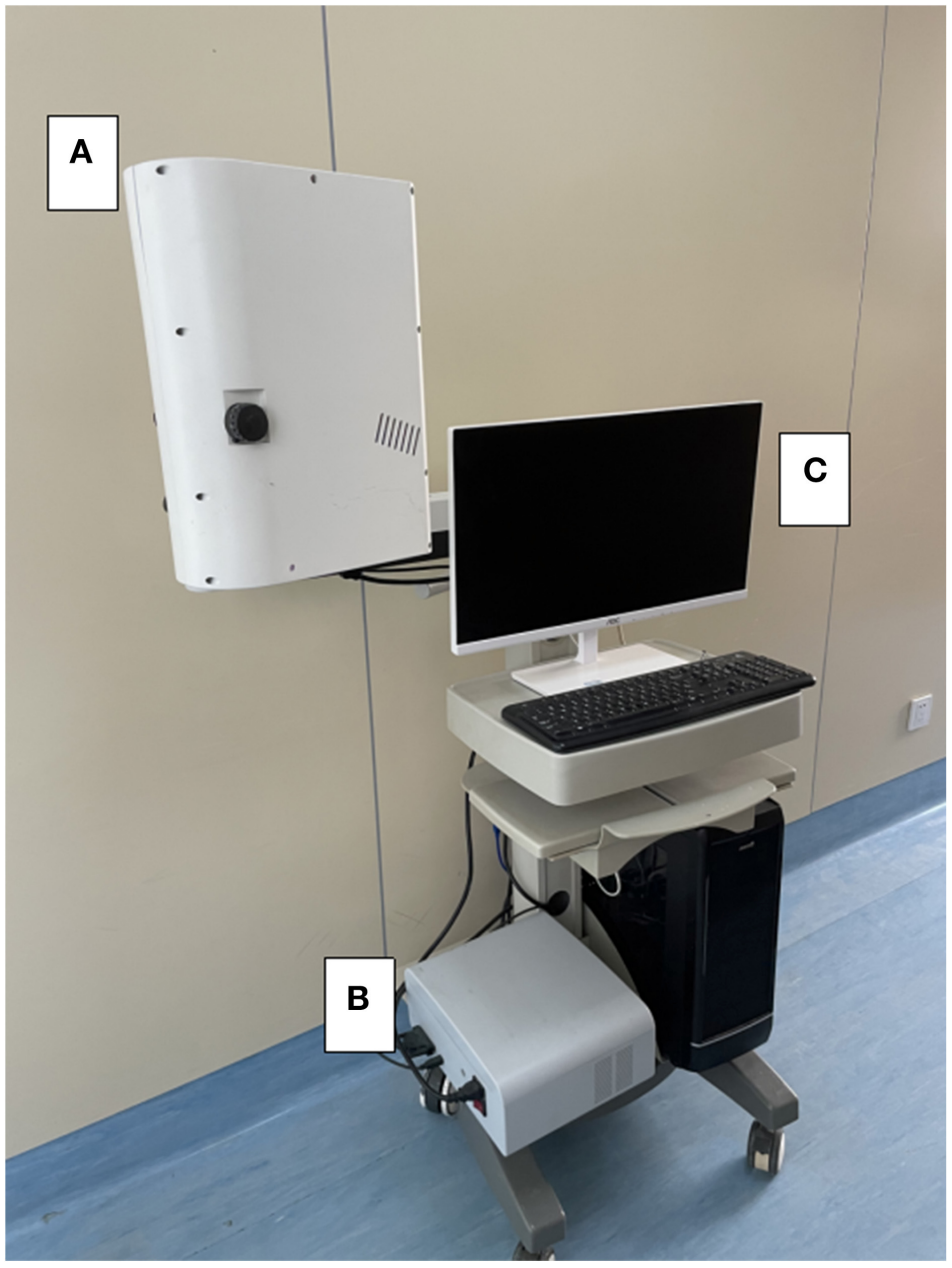
Device settings for intraoperative laser speckle imaging. In the intraoperative setting of the laser speckle device, the laser imager **(A)** is fixed on the clamped joint and connected to the mobile trolley, allowing free positioning in the surgical area. **(B)** The standard USB and FireWire interface can be connected to the laser light source. The computer **(C)** for data recording.

The laser imager (SIM BFI HR Pro, SIM Opto-Technology Co., Ltd, Wuhan, China, www.simopto.com) we used is conducted on a trolley by a rigid, extendable arm and attached with a mobile stand to allow versatile positioning over the interested imaging field. The imager was linked to a computer by a conventional universal serial bus (USB) and FireWire connections for data acquisition (Figure 1). The laser speckle imager was positioned 200 mm above and perpendicular to the exposed cortical region after craniotomy and durotomy. The laser penetration depth varies between 0.5 and 1 mm, depending on the optical qualities of the laser light and the sampled tissue (23, 26). The arbitrary perfusion unit CBF-Flux was used to record cortex perfusion using custom-designed data gathering software (SIM BFI measurement software, Version V2.0, SIMBFI HR PRO, CN). The mean flow in the observed region of interest (which can be any size or location) is computed and shown in real-time.

### Operation

**Figure 2:**
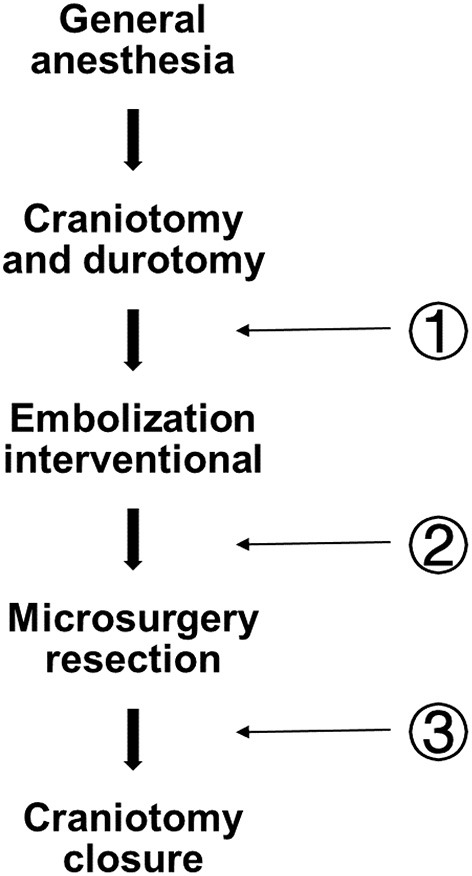
Flow diagram depicting the whole operational fixed procedure and three LSCI measurement sites. Pre-em (pre-embolization); Post-em (post-embolization); and Post(post-surgery).

With the development of biplane angiography suite operation, the number of patients treated with hybrid surgery for bAVM has increased. The angiography system includes a vertical monoplane detector, a ceiling-mounted one(UNIQ FD20/20, Philips, The Netherlands), and an operational microscope (Pentero, Carl Zeiss, Germany) in the operating room (10). Both bAVM resection and pre-surgical embolization were done at the one-stop stage in all. Under general anesthesia, endovascular embolization was conducted utilizing the biplane flat-panel angiographic suite with 3D reconstruction. Intraoperative DSA and LSCI are usually performed during the whole surgery (Figure 2).

Aseptic techniques were used throughout the procedure to provide a sterile environment. After craniotomy and durotomy, preoperative embolization was performed by using either n-butyl cyanoacrylate (Trufill, Cordis Neurovascular, Inc., Miami Lakes, FL, USA), ethylene-vinyl copolymer (Onyx 18 or 34, Medtronic, Inc., Minneapolis, Minnesota, USA). In hybrid surgery, surgical resection is performed without changing the patient's posture. After the excision of bAVM, DSA was used to evaluate the residual nidus. Arteriovenous malformations transit time (AVTT) was defined as the time required for blood to flow from the feeding artery to the drainage vein. During bAVM treatment, LSCI was performed at fixed at three measurement points (Figure 2): Pre-em(pre-embolization); Post-em(post-embolization); and Post(post-surgery).

### Statistical Analysis

Data are presented as mean ± SD. Statistics were generated by GraphPad Prism for Win (Version 8.0f, GraphPad Software, San Diego, CA, USA). Comparisons between two groups (survey scores) were achieved using the Student *t*-test. Two-tailed *p*-values < 0.05 were considered statistically significant.

## Results

### Patient Characteristic

Our study enrolled 14 patients with bAVM who were considered suitable for hybrid surgery under general anesthesia. The demographic characteristics of the patient population are listed in Table 1. A total of 10 men and 4 women were enrolled, and the mean age was 34 years (range 10–59 years). Out of these, eight patients were diagnosed with grade 4 Spetzler-Martin bAVM, five patients were diagnosed with grade 3 bAVM, and 1 was diagnosed with grade 5 bAVM. The clinical presentation was hemorrhage in 14.2% of patients, epilepsy in 42.9%, headache in 35.7% of patients, and neurological deficit in 7.2%.

**Figure 3:**
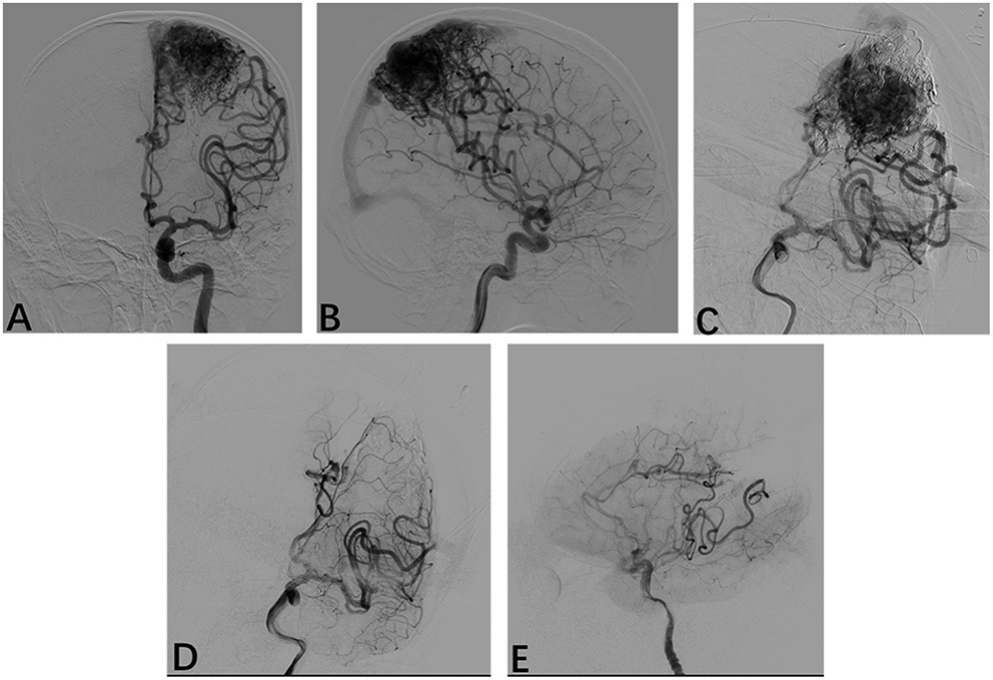
Radiological profiles of a male seizure patient. **(A,B)** Intraoperative angiography shows following injections of Onyx18 with partial obliteration of the bAVM; **(D,E)** Following resection, intraoperative anteroposterior and lateral projection angiograms show that the AVM has been obliterated.

A total of 42 times LSCI were performed. All patients underwent DSA after resection, which showed that bAVM had been completely removed. A typical example is shown in Figure 3. All patients underwent a CT scan within 24 h after the operation to check for bleeding. Postoperative bleeding occurred in two cases.

### AVTT During AVM Embolization

**Figure 4:**
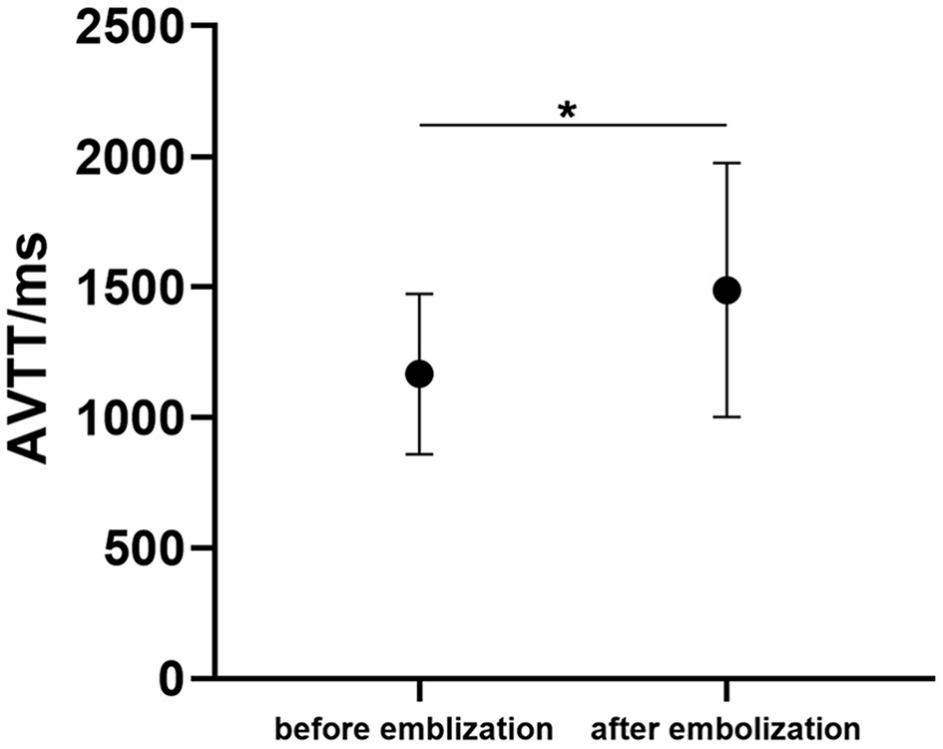
The arteriovenous malformations transit time (AVTT) before and after bAVM embolization. (^*^p < 0.05 for after-embolization).

In hybrid surgery, all 14 patients underwent DSA at least three times. The present study analyzed the AVTT of bAVM before and after embolization. AVTT before embolization (1,180.56 ± 329.20 ms) was significantly shorter than that after embolization (1,597.22 ± 429.11 ms, *p* < 0.05, Figure 4).

### Dynamics of LSCI During Perfusion Changes

**Figure 5:**
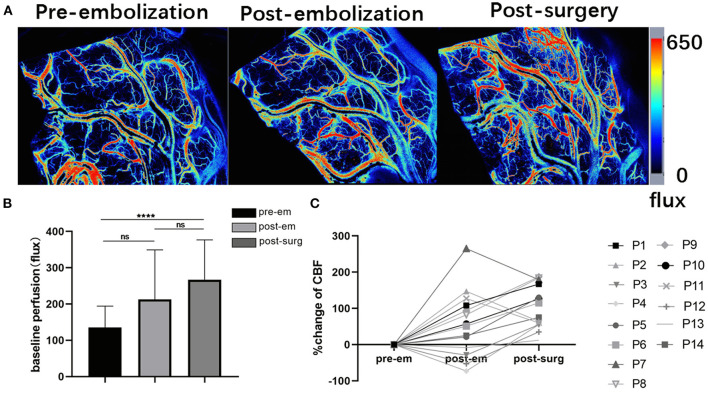
Intraoperative laser speckle contrast imaging's applicability **(A)**. Screenshots showing cortical baseline perfusion following craniotomy and durotomy immediately, after embolization, and after resection taken with intraoperative laser speckle imaging; **(B)** Pre-embolization, post-embolization, and post-surgery mean cortical baseline perfusion (CBF-Flux) in three stages (^****^p < 0.01 for post-surgery, *p* > 0.05 for post-embolization); **(C)** Cortex CBF reactivity following bAVM embolization and bAVM excision in 14 individuals undergoing hybrid surgery.

In each case, satisfactory CBF laser speckle patterns are obtained. This method accurately visualized blood flow in the cortical arteries surrounding the bAVM (Figure 5A).

Baseline perfusion in the cortex was (135 ± 59 flux) before bAVM embolization, and cerebral flow in the cortex increased after embolization (223 ± 136 flux), although these differences were not significant. After bAVM resection, the perfusion was (267 ± 109 flux), and the cortical CBF flux increased significantly compared with that before embolization (*p* < 0.001, Figure 5B). In 14 patients who underwent hybrid surgery for bAVM, we could detect flow fluctuations in cortical blood flow during surgery (Figure 5C).

## Discussion

Hybrid surgery (microsurgical resection coupled with endovascular embolization) for bAVM has been proved to be safe and effective, with high rates of immediate, complete, and permanent bAVM obliteration (5, 6, 10, 12, 13, 27). Routine intraoperative DSA after microsurgical resection can identify the AVM remnant directly for one-stop resection (12, 27). Preoperative partial embolization of the malformation can assist in surgical positioning and decrease blood flow of the malformation, which contributes to minimizing the risk of intraoperative bleeding (28–31). However, after bAVM resection, excessive perfusion of adjacent cortical tissue and pathological increase of local intravascular pressure (32–34), may generate cerebral hemorrhage or edema, which worsens the postoperative course. Two possible causes for the hyperperfusion were proposed: (1) the microcirculation in the AVM area could not protect itself from the rapid increase of pressure from low to normal through automatic regulation reaction, resulting in the breakthrough of normal perfusion pressure (NPPB). This hypothesis is supported by the fact that there are significant differences between perinidal capillaries and other capillaries in the brain, which helps to prove that perinidal capillaries may be apt to rupture once bAVM is removed (5, 32). (2)Occlusive hyperemia, the outcome of propagated thromboembolism in a dilated venous drainage system that became superfluous after AVM excision is occlusive hyperemia (35).

Our study suggested that the severity of postoperative hyperperfusion in the brain surrounding the resected AVM is locally distinct. Furthermore, the density of the cortical layer around vascular malformation was higher than that of normal cortical vessels. Asgari et al. (8) showed the removal of bAVM usually led to significant increases of SaO_2_ in the cortical area around malformation, which indicated that the brain would suffer from congestive injury, primarily due to the excessive inflow of capillaries, which was used to be an ischemic area before the removal of bAVM.

The applicability of dynamic and noninvasive real-time monitoring of CBF in patients undergoing bAVM surgery by LSCI has been proven in our research, which involves a series of CBF changes related to bAVM resection. LSCI has been proved to be able to intuitively, effectively, and accurately reflect the changes in hemodynamics. The operation protocols were well managed in all the patients, and no adverse effects were found. Patient safety and image quality were not compromised by repeated measurements.

Intraoperative monitoring enhances the effectiveness of neurosurgery and the safety of patients. CBF remains the most important driver of tissue viability. However, there are no reliable means to evaluate it at present. Although many imaging methods for studying cerebral hemodynamics have been reported, such as positron emission tomography (PET), SPECT, orthogonal polarization spectral (OPS) imaging, dynamic perfusion CT, or ICG-FLOW800 perfusion study (8, 17, 21, 36, 37), their intraoperative application is limited by immobilization, invasiveness, high financial, and personal costs.

Based on dynamic light scattering off moving particles, LSCI works as an optical blood flow visualization and quantification technique (erythrocytes). The camera records the speckle pattern, and the software computes the contrast of each pixel to create a color-coded map of CBF (20). At present, no data exists for direct cortex blood flow evaluation in patient with bAVM. LSCI has been used during intracranial surgery to map the motor cortex in awake craniotomy, identify cortical regions at risk of infarction after decompressive craniectomy, and show increased blood flow after arterial anastomosis (20, 38, 39). Through direct assessment of tissue perfusion, LSCI provides immediate functional feedback on cortical blood flow (20). Previously, Takeshita et al. (40) reported the value of SPECT in monitoring the changes in regional CBF after the removal of bAVM. They believe that SPECT plays a significant role in detecting the hyperperfusion of the area adjacent to the malformation. However, routine assessment of CBF using SPET is theoretically and financially difficult and has not been widely used. LSCI may be a non-invasive, cost-effective, and convenient method to detect the changes in normal tissue perfusion after resection, with high temporal and spatial resolution.

This present study found that the cortical flow index following bAVM removal is significantly higher than it was preoperatively. This may imply altered hemodynamics during bAVM surgery, leading to the redistribution of blood flow into the capillaries of the surrounding cortex. In our data, three patients had a reduction in flux following embolization, as shown in Figure 5C. This may result from our surgical strategy. First, the target of embolization we chose might not be the main feeding artery of bAVM, but a tiny feeding artery. Therefore, there was no obvious change in perfusion of the cortex around the nidus. Additionally, when we finished embolization, the blood vessels experience vasospasm due to vessel “stimulation” caused by catheter manipulation during embolization. Although usually asymptomatic, it can lead to decreased blood flow and potentially poor recanalization in the region of the vasospasm (41).

Conger et al. (29) and Donzelli et al. (1) reported that preoperative embolization could reduce the size and grade of the bAVM so that patients with higher-grade lesions were able to be treated with curative microsurgery, thereby minimizing complications and improving outcomes. Our study showed that the transit time of bAVM is significantly longer after endovascular embolization than preoperative, implying that endovascular embolization decreases the nidus flow velocity, and minimizes the risk of intraoperative bleeding in microsurgery resection of high-grade bAVM. However, in the treatment of high-grade bAVM, especially Spetzler-Martin 5 bAVM, we need to pay more attention to the occurrence of hyperperfusion. Once we block the main feeding artery and partial nidus occlusion, there will be high perfusion in the area adjacent to bAVM. The increase in blood flow might be the initiator of intraoperative and postoperative bleeding. In this “outlier” patient, blood loss in microsurgery resection might decrease the “flux” value in CBF around malformation (Figure 5C).

Treatment of bAVM, including high-grade bAVM, requires hemodynamic evaluation (5). Previous studies (9, 17, 40, 42) suggested cerebral perfusion harassment by preoperative embolism could be measured with DSA, SPECT, or iFlow (parametrically color-coded angiography), which cannot instantly evaluate the flow dynamic and normal cortex perfusion. In our hybrid-OR, we manage endovascular treatment and microsurgical resection in one-stop. To ensure surgical safety and effectiveness, we routinely utilize intraoperative DSA and LSCI to monitor bAVM hemodynamic changes in real-time and prevent intraoperative bleeding. That could further help to reduce surgical complications and improve long-term outcomes.

Our research has some limitations. First, the sample size of surgical patients is not large enough. We need more clinical data to reveal the usefulness of LSCI in monitoring cerebral hemodynamic change. Second, due to the limited depth of tissue penetration of LSCI, they may only evaluate superficial cortical perfusion. Furthermore, since the technology we used has not been incorporated into the surgical microscope, the operation must be paused while the LSCI device is positioned over the surgical field during the whole measurement interval.

## Conclusion

The combination of embolization and microsurgical resection in hybrid surgery might be a safe and successful method for the treatment of bAVM. With high spatial-temporal resolution and good image quality, LSCI enables real-time dynamic imaging of blood flow within the cortical vasculature and assessment of cortical perfusion around the AVM at a low cost. LSCI can be used as a supplement for intraoperative CBF dynamic imaging to improve the quality of neurovascular treatment.

## Data Availability Statement

The original contributions presented in the study are included in the article/supplementary material, further inquiries can be directed to the corresponding author/s.

## Ethics Statement

This study was obtained from the Zhongnan Hospital of Wuhan University Ethics Committee (Kelun-2017005). Written informed consent to participate in this study was provided by the participants' legal guardian/next of kin.

## Author Contributions

JC: conception and design of the study. TZ, ST, and KZ: data acquisition. ST, XL, and KZ: software. ST and YF: analysis and interpretation of the data. WZ, ST, YF, and JC: co-drafted the manuscript. All authors read and approved thefinal manuscript.

## Conflict of Interest

The authors declare that the research was conducted in the absence of any commercial or financial relationships that could be construed as a potential conflict of interest.

## Publisher's Note

All claims expressed in this article are solely those of the authors and do not necessarily represent those of their affiliated organizations, or those of the publisher, the editors and the reviewers. Any product that may be evaluated in this article, or claim that may be made by its manufacturer, is not guaranteed or endorsed by the publisher.

## Abbreviations

bAVM: brain arteriovenous malformations
ICH: intracerebral hemorrhage
DSA: digital subtraction angiography
CBF: cerebral blood flow
LSCI: laser speckle-contrast imaging
AVTT: arteriovenous malformations transit time
NPPB: normal perfusion pressure breakthrough
ICG: indocyanine green videoangiography
hybrid-OR: hybrid operating room.

## References

[1] Donzelli GF Nelson J McCoy D McCulloch CE Hetts SW Amans MR. The effect of preoperative embolization and flow dynamics on resection of brain arteriovenous malformations. J Neurosurg. (2019) 132:1836–44. 10.3171/2019.2.JNS182743 31100732 PMC6858934

[2] Lawton MT Rutledge WC Kim H Stapf C Whitehead KJ Li DY. Brain arteriovenous malformations. Nat Rev Dis Primers. (2015) 1:15008. 10.1038/nrdp.2015.8 27188382

[3] Luksik AS Law J Yang W Garzon-Muvdi T Caplan JM Colby G. Assessing the Role of Preoperative Embolization in the Surgical Management of Cerebral Arteriovenous Malformations. World Neurosurg. (2017) 104:430–41. 10.1016/j.wneu.2017.05.026 28512050

[4] Antkowiak L Putz M Rogalska M Mandera M. Multimodal treatment of pediatric ruptured brain arteriovenous malformations: a single-center study. Children (Basel). (2021) 8:215. 10.3390/children8030215 33799749 PMC7998913

[5] Ye X Wang L Li MT Chen XL Wang H Ma L. Hemodynamic changes in superficial arteriovenous malformation surgery measured by intraoperative ICG fluorescence videoangiography with FLOW 800 software. Chin Neurosurg J. (2020) 6:29. 10.1186/s41016-020-00208-y 32922958 PMC7416385

[6] Gruter BE Mendelowitsch I Diepers M Remonda L Fandino J Marbacher S. Combined endovascular and microsurgical treatment of arteriovenous malformations in the hybrid operating room. World Neurosurg. (2018) 117:e204–14. 10.1016/j.wneu.2018.05.241 29890278

[7] Starke RM Komotar RJ Hwang BY Fischer LE Garrett MC Otten ML. Treatment guidelines for cerebral arteriovenous malformation microsurgery. Br J Neurosurg. (2009) 23:376–86. 10.1080/02688690902977662 19637008

[8] Asgari S Rohrborn HJ Engelhorn T Fauser B Stolke D. Intraoperative measurement of cortical oxygen saturation and blood volume adjacent to cerebral arteriovenous malformations using near-infrared spectroscopy. Neurosurgery. (2003) 52:1298–304. 10.1227/01.NEU.0000064801.78895.86 12762875

[9] Durner G Wahler H Braun M Kapapa T Wirtz CR König R. The value of intraoperative angiography in the time of indocyanine green videoangiography in the treatment of cerebrovascular lesions: efficacy, workflow, risk-benefit and cost analysis A prospective study. Clin Neurol Neurosurg. (2021) 205:106628. 10.1016/j.clineuro.2021.106628 33895619

[10] Yihui M Lesheng W Jichun S Lixin D Tingbao Z Yu F. Single-center retrospective analysis of one-stop hybrid surgery for brain rteriovenous malformations. Res Squ. (2021). 10.21203/rs.3.rs-908476/v2

[11] Katz JM Gologorsky Y Tsiouris AJ Wells-Roth D Mascitelli J Gobin YP. Is routine intraoperative angiography in the surgical treatment of cerebral aneurysms justified? A consecutive series of 147 aneurysms. Neurosurgery. (2006) 58:719–27. 10.1227/01.NEU.0000204316.49796.A3 16575336

[12] Murayama Y Arakawa H Ishibashi T Kawamura D Ebara M Irie K. Combined surgical and endovascular treatment of complex cerebrovascular diseases in the hybrid operating room. J Neurointerv Surg. (2013) 5:489–93. 10.1136/neurintsurg-2012-010382 22661589

[13] Kotowski M Sarrafzadeh A Schatlo B Boex C Narata AP Pereira VM. Intraoperative angiography reloaded: a new hybrid operating theater for combined endovascular and surgical treatment of cerebral arteriovenous malformations: a pilot study on 25 patients. Acta Neurochir (Wien). (2013) 155:2071–8. 10.1007/s00701-013-1873-z 24036674

[14] Wang A Mandigo GK Feldstein NA Sisti MB Connolly ES Solomon RA. Curative treatment for low-grade arteriovenous malformations. J Neurointerv Surg. (2020) 12:48–54. 10.1136/neurintsurg-2019-015115 31300533

[15] Markl M Wu C Hurley MC Ansari SA Carroll TJ Rahme RJ. Cerebral arteriovenous malformation: complex 3D hemodynamics and 3D blood flow alterations during staged embolization. J Magn Reson Imaging. (2013) 38:946–50. 10.1002/jmri.24261 24027116

[16] Acerbi F Vetrano IG Sattin T Falco J de Laurentis C Zattra CM. Use of ICG videoangiography and FLOW 800 analysis to identify the patient-specific venous circulation and predict the effect of venous sacrifice: a retrospective study of 172 patients. Neurosurg Focus. (2018) 45:E7. 10.3171/2018.4.FOCUS18120 29961380

[17] Faber F Thon N Fesl G Rachinger W Guckler R Tonn JC. Enhanced analysis of intracerebral arterioveneous malformations by the intraoperative use of analytical indocyanine green videoangiography: technical note. Acta Neurochir (Wien). (2011) 153:2181–7. 10.1007/s00701-011-1141-z 21894496

[18] Bilbao CJ Bhalla T Dalal S Patel H Dehdashti AR. Comparison of indocyanine green fluorescent angiography to digital subtraction angiography in brain arteriovenous malformation surgery. Acta Neurochir (Wien). (2015) 157:351–9. 10.1007/s00701-014-2287-2 25488175

[19] Hecht N Woitzik J Dreier JP Vajkoczy P. Intraoperative monitoring of cerebral blood flow by laser speckle contrast analysis. Neurosurg Focus. (2009) 27:E11. 10.3171/2009.8.FOCUS09148 19795950

[20] Hecht N Woitzik J König S Horn P Vajkoczy P. Laser speckle imaging allows real-time intraoperative blood flow assessment during neurosurgical procedures. J Cereb Blood Flow Metab. (2013) 33:1000–7. 10.1038/jcbfm.2013.42 23512134 PMC3705427

[21] Kawamata T Kawashima A Yamaguchi K Hori T Okada Y. Usefulness of intraoperative laser Doppler flowmetry and thermography to predict a risk of postoperative hyperperfusion after superficial temporal artery-middle cerebral artery bypass for moyamoya disease. Neurosurg Rev. (2011) 34:355–62. 10.1007/s10143-011-0331-8 21643682

[22] Richards LM Towle EL Fox DJ Dunn AK. Intraoperative laser speckle contrast imaging with retrospective motion correction for quantitative assessment of cerebral blood flow. Neurophotonics. (2014) 1:015006. 10.1117/1.NPh.1.1.015006 26157974 PMC4479045

[23] Senarathna J Rege A Li N Thakor NV. Laser Speckle Contrast Imaging: theory, instrumentation and applications. IEEE Rev Biomed Eng. (2013) 6:99–110. 10.1109/RBME.2013.2243140 23372086

[24] Vaz PG Humeau-Heurtier A Figueiras E Correia C Cardoso J. Laser Speckle Imaging to Monitor Microvascular Blood Flow: A Review. IEEE Rev Biomed Eng. (2016) 9:106–20. 10.1109/RBME.2016.2532598 26929060

[25] Dunn AK. Laser speckle contrast imaging of cerebral blood flow. Ann Biomed Eng. (2012) 40:367–77. 10.1007/s10439-011-0469-0 22109805 PMC3288249

[26] Winship IR. Laser speckle contrast imaging to measure changes in cerebral blood flow. Methods Mol Biol. (2014) 1135:223–35. 10.1007/978-1-4939-0320-7_19 24510868

[27] Choi E Lee JY Jeon HJ Cho BM Yoon DY. A hybrid operating room for combined surgical and endovascular procedures for cerebrovascular diseases: a clinical experience at a single centre. Br J Neurosurg. (2019) 33:490–4. 10.1080/02688697.2019.1617403 31092005

[28] Sato K Matsumoto Y Tominaga T Satow T Iihara K Sakai N. Complications of endovascular treatments for brain arteriovenous malformations: a nationwide surveillance. AJNR Am J Neuroradiol. (2020) 41:669–75. 10.3174/ajnr.A6470 32193193 PMC7144660

[29] Conger A Kulwin C Lawton MT Cohen-Gadol AA. Endovascular and microsurgical treatment of cerebral arteriovenous malformations: current recommendations. Surg Neurol Int. (2015) 6:39. 10.4103/2152-7806.153707 25883831 PMC4392538

[30] Rutledge C Cooke DL Hetts SW Abla AA. Brain arteriovenous malformations. Handb Clin Neurol. (2021) 176:171–8. 10.1016/B978-0-444-64034-5.00020-1 33272394

[31] Feng AY Sussman ES Jin MC Wong S Lopez J Pulli B. Intraoperative Neuromonitoring for cerebral arteriovenous malformation embolization: a propensity-score matched retrospective database study. Cureus. (2021) 13:e12946. 10.7759/cureus.12946 33654622 PMC7910512

[32] Kato Y Sano H Nonomura K Kanno T Katada K Takeshita G. Normal perfusion pressure breakthrough syndrome in giant arteriovenous malformations. Neurol Res. (1997) 19:117–23. 10.1080/01616412.1997.11740783 9175138

[33] Rangel-Castilla L Spetzler RF Nakaji P. Normal perfusion pressure breakthrough theory: a reappraisal after 35 years. Neurosurg Rev. (2015) 38:399–404. 10.1007/s10143-014-0600-4 25483235

[34] Mansour A Rashad S Niizuma K Fujimura M Tominaga T. A novel model of cerebral hyperperfusion with blood-brain barrier breakdown, white matter injury, and cognitive dysfunction. J Neurosurg. (2019) 1–13. 10.3171/2019.7.JNS19212 31628277

[35] Al-Rodhan NR Sundt TM Piepgras DG Nichols DA Rüfenacht D Stevens LN. Occlusive hyperemia: a theory for the hemodynamic complications following resection of intracerebral arteriovenous malformations. J Neurosurg. (1993) 78:167–75. 10.3171/jns.1993.78.2.0167 8421198

[36] Ul'yanov SS Tuchin VV Bednov AA Brill GE Zakharova EI. The application of speckle interferometry for the monitoring of blood and lymph flow in microvessels. Lasers Med Sci. (1997) 12:31–41. 10.1007/BF02763919

[37] Pennings FA Ince C Bouma GJ. Continuous real-time visualization of the human cerebral microcirculation during avm surgery using orthogonal polarization spectral imaging. Neurosurgery. (2006) 59:167–71. 10.1227/01.NEU.0000219242.92669.3B 28180616

[38] Gallagher MJ Hogg FRA Zoumprouli A Papadopoulos MC Saadoun S. Spinal cord blood flow in patients with acute spinal cord injuries. J Neurotrauma. (2019) 36:919–29. 10.1089/neu.2018.5961 30351245

[39] Klijn E Hulscher HC Balvers RK Holland WP Bakker J Vincent AJ. Laser speckle imaging identification of increases in cortical microcirculatory blood flow induced by motor activity during awake craniotomy: Clinical article. J Neurosurg. (2013) 118:280–6. 10.3171/2012.10.JNS1219 23176333

[40] Takeshita G Toyama H Nakane K Nomura M Osawa H Ogura Y. Evaluation of regional cerebral blood flow changes on perifocal brain tissue SPECT before and after removal of arteriovenous malformations. Nuclear Med Commun. (1994) 15:461 10.1097/00006231-199406000-00011 8078643

[41] Balami JS White PM McMeekin PJ Ford GA Buchan AM. Complications of endovascular treatment for acute ischemic stroke: Prevention and management. Int J Stroke. (2018) 13:348–61. 10.1177/1747493017743051 29171362

[42] Shellikeri S Bai H Setser RM Hurst RW Cahill AM. Association of intracranial arteriovenous malformation embolization with more rapid rate of perfusion in the peri-nidal region on color-coded quantitative digital subtraction angiography. J Neurointerv Surg. (2020) 12:902–5. 10.1136/neurintsurg-2019-015776 32188762

